# Force-Triggered Thermodynamically Uphill Disulfide Reduction through Sulfur Oxidation State Control

**DOI:** 10.1021/jacs.5c13084

**Published:** 2025-10-01

**Authors:** Marc Mora, Georgia Cohen, William Cranton, Olaia Anton, Amy E. M. Beedle, Guillaume Stirnemann, Sergi Garcia-Manyes

**Affiliations:** † Department of Physics, Randall Centre for Cell and Molecular Biophysics, Centre for the Physical Science of Life and London Centre for Nanotechnology, King’s College London, Strand, London WC2R 2LS, U.K.; ‡ Single Molecule Mechanobiology Laboratory, The Francis Crick Institute, 1 Midland Road, London NW1 1AT, U.K.; § Department of Physics and Centre for the Physical Science of Life, King’s College London, Strand, London WC2R 2LS, U.K.; ∥ CPCV, Département de Chimie, École Normale Supérieure, PSL University, Sorbonne University, CNRS, 75005 Paris France

## Abstract

In addition to thermal energy, current, and light, mechanical forces activate chemical reactions, often steering reaction pathways that result in products different from those obtained under thermodynamic control. Single-molecule mechanochemistry experiments have probed how the forced activation of a single covalent bond results in accelerated scission of both homolytic and heterolytic bonds, and the ring-opening of strained mechanophores in long polymers. Due to its mechanistic simplicity, the concerted S_N_2 thiol–disulfide nucleophilic substitution has been successfully used as a model system to interrogate how the nucleophilicity of an attacking organic, low-oxidation state thiol determines the force dependency of the thiol/disulfide exchange rate. Inorganic sulfur-oxyanions are comparatively much less reactive. Whether mechanical forces can activate the rupture of a protein disulfide by sulfur-oxyanions featuring higher oxidation states remains unknown. Here we employ single-molecule force-clamp spectroscopy, complemented by density functional theory (DFT) calculations and colorimetric assay measurements, to show that the thermodynamically nonfavored reduction of a disulfide bond by inorganic oxyanions can be activated by mechanical force. Occurring within the core of a protein with a physiological mechanical role, the force-unlocked reactivity has a direct impact on protein elasticity.

## Graphical abstract

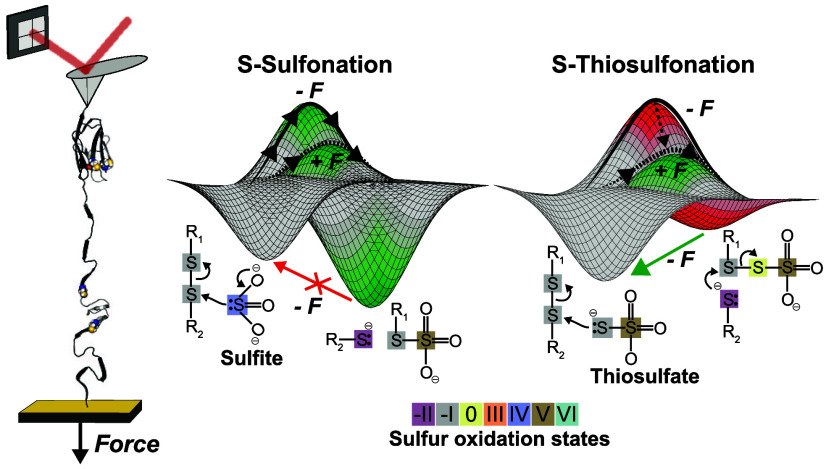

## Introduction

Mechanical force provides an energy source, alternative to heat, electric current and light, to initiate a chemical reaction.^1^ Specifically, externally applied force can bias the potential energy surface of chemical reactions, favoring routes to products that are not necessarily populated according to a Boltzmann distribution of energy barriers.^2^ For example, the application of forces through ultrasounds catalyzed covalent ring-opening reactions in mechanophores contained in long polymer chains^3^ and biased reaction pathways toward the formation of thermodynamically disfavored isomers.^4^ In some instances, reaching low-probability products resulted from visiting transition state structures the lifetime of which was regulated by force.^5^

Single-molecule experiments have provided detailed mechanistic understanding of the localized effect of force on the activation of a specific covalent bond^6^ for a variety of homolytic and heterolytic reactions.^7^ In particular, force-clamp experiments demonstrated how the bimolecular nucleophilic substitution leading to disulfide rupture^8^ is accelerated by force,^9,10^ and showed how the chemical nature of the attacking nucleophile regulates the rate of disulfide reduction.^11–13^ For the specific case of organic thiols (−II sulfur oxidation state), a direct correlation between the electrostatic partial charge localized on the nucleophilic sulfur and the measured disulfide reduction rate was obtained.^14,15^ However, a systematic understanding of how force modulates the rate and outcome of bimolecular chemical reactions that encompass a broad range of oxidation states is missing.

Due to its unique electronic configuration, displaying an empty external d-orbital available for bonding ([Ne]­3s^2^3p^4^3d^0^), sulfur exhibits versatile reactivity with oxidation states ranging from −II to +VI. Hence, inorganic sulfur nucleophiles, exhibiting a broad range of oxidation states, provide an excellent platform to interrogate how force modulates the underlying one-dimensional (1D) energy landscape of S_N_2 reactions, potentially affecting both their kinetics and thermodynamics.

## Results

We started by testing the reactivity of sulfite (SO_3_^2–^), which exhibits a +IV sulfur oxidation state (Figure 1A). In the absence of force, sulfite reduced the symmetric disulfide bridge of the Ellman’s reagent^16^ (Figure 1B). These findings were corroborated with density functional theory (DFT) calculations (using the M06–2X^17^ functional with the ma-def2-TZVP basis set^18^ in CPCM implicit solvent) to predict the standard Gibbs free energy (Δ*G* ^0^) associated with the cleavage of the disulfide bond between two model thiols (here, methyl thiols) by sulfite, displaying a favorable tendency toward disulfide bridge reduction (Δ*G* ^0^ = −7.6 kcal mol^–1^), Figure 1C. From the kinetic perspective, however, the localized charge on the sulfur estimated based on the DFT calculations is only slightly negative (−0.055 e, Figure S1)—suggesting a much slower reactivity than that measured for organic (−II) thiols, for which we could previously establish a correlation between the rate constant and the sulfur charge.^15^ A different combination of DFT functional and basis set gave the same qualitative results (Figure S1). While recent machine-learned reactive potentials^19^ applied to chemical reactivity^20^ would provide a more quantitative assessment of disulfide bond reactivity toward these nucleophiles (for example by taking into account full explicit solvation and the presence of ions), our admittedly simpler calculations already capture our experimental observations.

**Figure 1:**
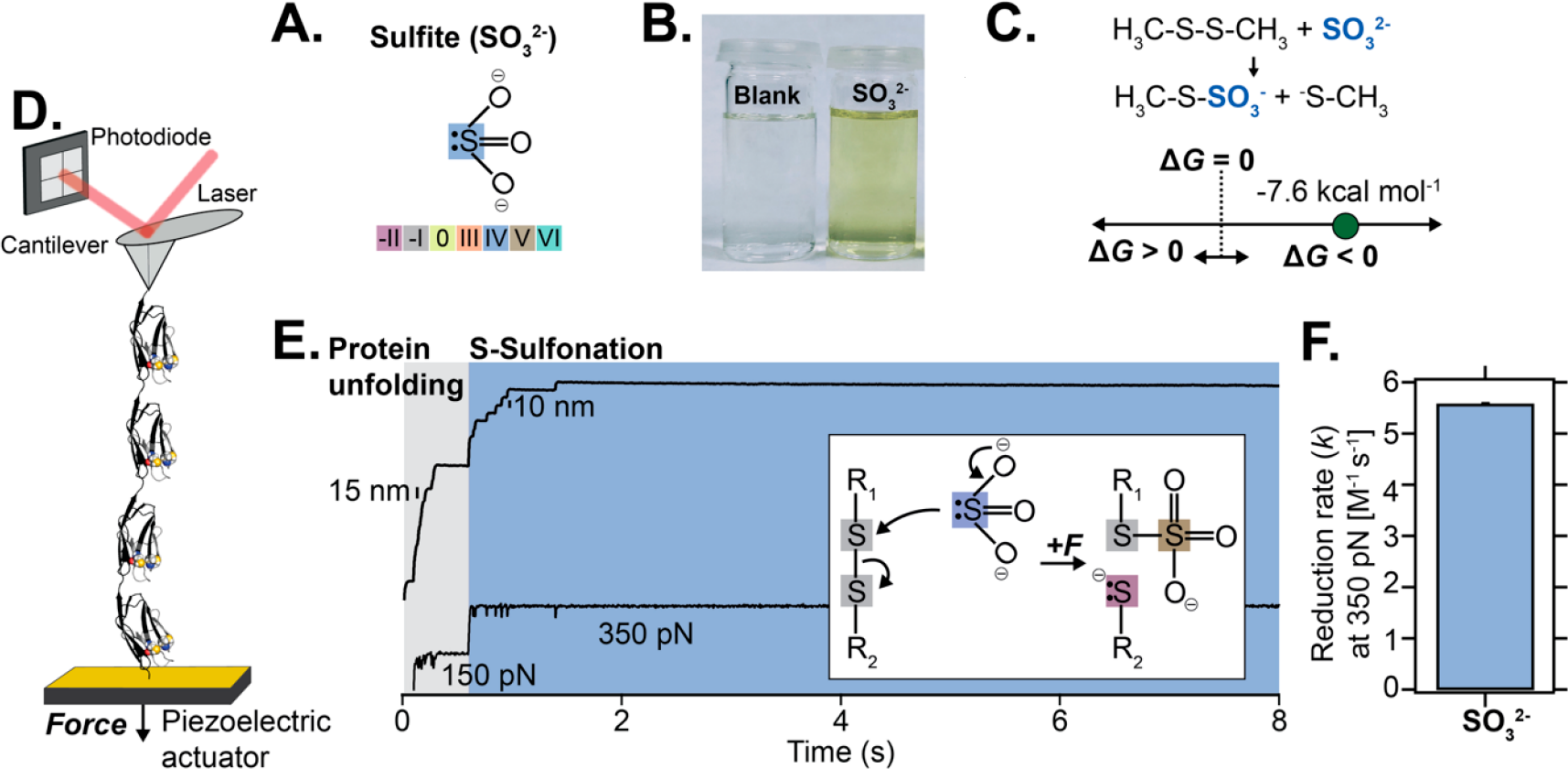
Force accelerates the thermodynamically spontaneous reduction of a protein disulfide bond by sulfite anion. (A) Sulfite anion and the sulfur oxidation state. (B) Sulfite disrupts the symmetric disulfide bridge of the Ellman’s reagent (*A*_λ=412 nm_ = 1.058 au). (C) DFT calculations of the standard free energy (M06–2X functional with the ma-def2-TZVP basis set in CPCM implicit solvent) associated with disulfide rupture of two methyl thiols by sulfite. (D) Schematic representation of the single-molecule force-clamp experiment, where an individual (Ig27_E24C–K55C_)_8_ polyprotein is stretched in an AFM. (E) Force application first triggers the unfolding of the protein up to the disulfide bond (gray region). Sulfite (250 mM, pH 7.5) induces the rupture of the stretched disulfide bond, marked by a ∼10 nm stepwise increase in the protein length (blue region). Inset: schematics of the sulfite-mediated disulfide rupture. (F) The concentration-normalized rate of protein disulfide rupture by sulfite at a constant force (*F* = 350 pN), pH 7.5; *N* = 15 reduction trajectories.

We then questioned whether mechanical force might accelerate the sulfite-mediated rupture of an individual disulfide bond. We employed a single-molecule force-clamp AFM assay designed earlier^9,12,15^ using a polyprotein containing eight repeats of a titin Ig27 mutant with a buried engineered disulfide bridge between positions 24 and 55, E24C–K55C (Figure 1D). Briefly, the application of a constant force (150 pN for 0.5 s) promoted the unfolding of the protein domain(s) up to the rigid disulfide bond, hallmarked by a ∼15 nm stepwise increase of the protein (Figure 1E). Each unfolding event readily exposes the previously cryptic disulfide bridge to the solution. Subsequently, the application of a higher force (*F* = 350 pN) in the presence of 250 mM sulfite at pH 7.5 triggered the cleavage of the disulfide bridge, fingerprinted by individual ∼10 nm steps that correspond to the length release of the amino acids previously trapped by the covalent disulfide bond. Averaging a number of independent reduction trajectories normalized by the sulfite concentration yielded a reduction rate of *k* = 5.58 M^–1^ s^–1^ (Figure 1F), which is certainly much slower than that measured for organic thiols with lower (−II) oxidation state,^15^ even though sulfite nucleophilicity (and the overall reaction thermodynamics) is modulated by its protonation state (p*k*_a_ = 6.97) (Figures S2–S4). Our results highlight that force can significantly accelerate the otherwise kinetically slow (yet thermodynamically favored) S_N_2 cleavage of a protein disulfide bond by sulfite.

We then queried whether a structurally similar compound such as thiosulfate (S_2_O_3_^2–^) (Figure 2A), with a nucleophilic sulfur atom with a lower oxidation state (−I)^21^ exhibits different reactivity toward disulfide reduction under force. Critically, both the Ellman’s assay (Figure 2B) and DFT calculations (Δ*G* ^0^ = +11.9 kcal mol^–1^) (Figure 2C) concluded that disulfide bond reduction by thiosulfate is thermodynamically nonfavored (which is qualitatively confirmed using another combination of DFT functional/basis set, Figure S5). We therefore questioned if the application of mechanical force would enable disulfide bond cleavage, even in these nonfavorable thermodynamic conditions. Our force-clamp experiments (Figure 2D) revealed that, albeit exhibiting a slower normalized rate at 350 pN of disulfide reduction (*k* = 0.38 M^–1^ s^–1^) than sulfite, thiosulfate (250 mM, pH 7.5) can indeed induce the nonspontaneous rupture of a disulfide bond that is exposed to a persistent and calibrated stretching force (Figure 2E). To elucidate how the kinetics of disulfide bond scission depends on the pulling force, we measured the time-course of disulfide bond rupture at several forces spanning 250 pN–450 pN (Figure 3A). To obtain the rate of reduction at any particular force, we averaged and normalized 16–40 individual traces, such as those shown in Figure 3A, and fitted the average trajectory to a single exponential (dotted lines in Figure 3B). From the exponential time constant of the fits (τ_R_) we calculated the rate of reduction as *r* = 1/τ_R_. The standard error of the mean (s.e.m.) of these data was estimated by bootstrapping. We then fitted the force dependency of the rate of reduction using the simple Arrhenius term *r*(*F*) = *r*_*o*_exp­(*F*Δ*x* ^‡^/*kT*), where *r*_0_ is the rate constant in the absence of force,^22^ yielding *r*_0_ = 0.0025 ± 0.0007 s^–1^ and Δ*x* ^‡^ = 0.42 ± 0.03 Å (Figure 3C). These results conclude that force accelerates the rate of disulfide bond reduction in the presence of a thermodynamically nonfavored nucleophile such as thiosulfate.

**Figure 2:**
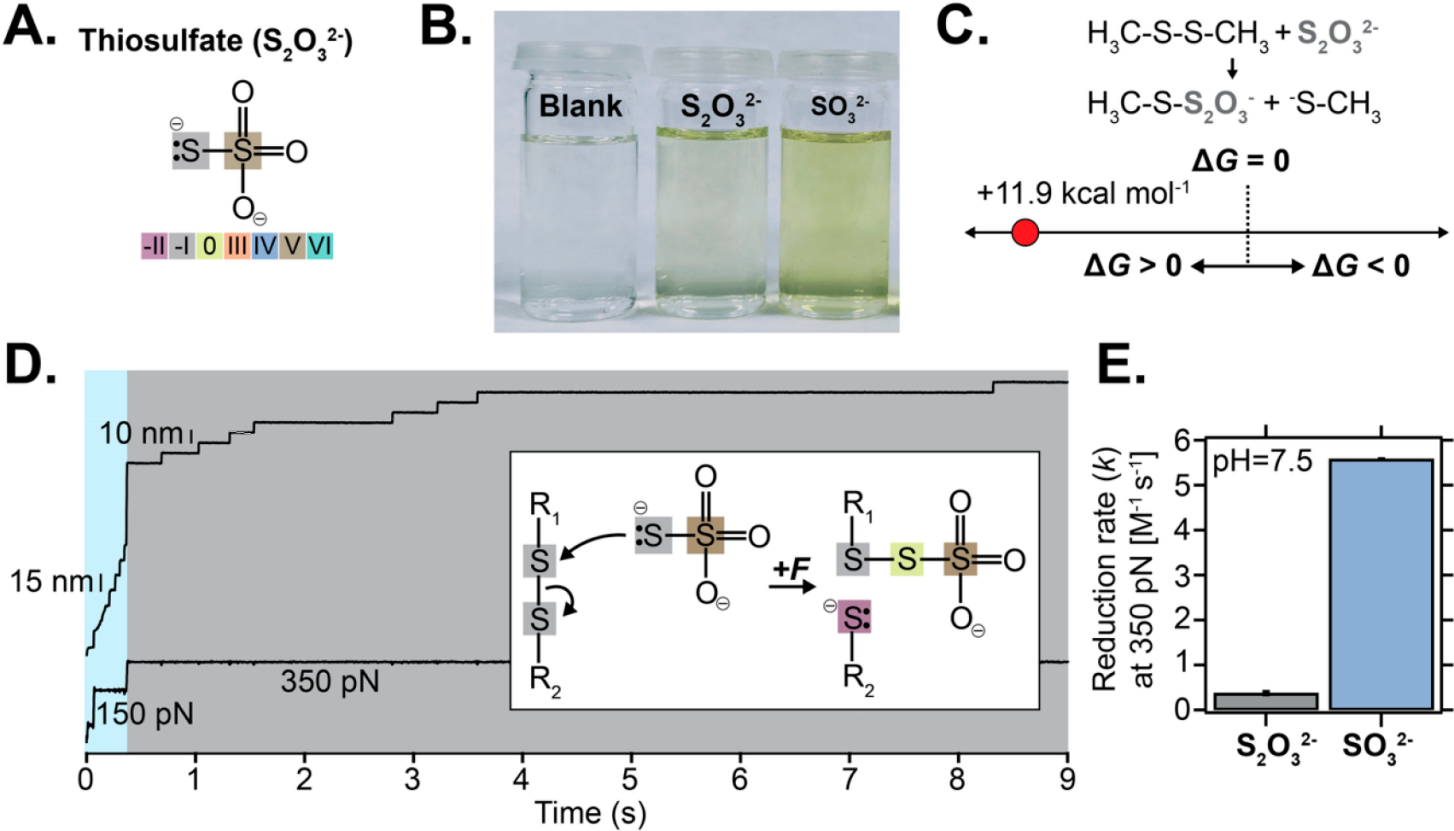
Mechanical force induces the thermodynamically impaired reduction of a protein disulfide bond by thiosulfate anion. (A) Chemical representation of thiosulfate anion. (B) Thiosulfate can barely react with the Ellman’s reagent, as opposed to sulfite (*A*_λ=412 nm_ = 0.270 au and 1.058 au, respectively). (C) DFT calculations of the standard free energy (M06–2X functional with the ma-def2-TZVP basis set in CPCM implicit solvent) associated with disulfide rupture of two methyl thiols by thiosulfate. (D) Kinetics of disulfide bond reduction at 350 pN in the presence of 250 mM of thiosulfate (pH 7.5). Inset: schematics of the underpinning chemical reactivity. (E) The concentration-normalized rate of protein disulfide rupture by thiosulfate (gray) and sulfite (blue, for comparison) at a constant force (*F* = 350 pN), pH 7.5; *N* = 34 trajectories.

**Figure 3:**
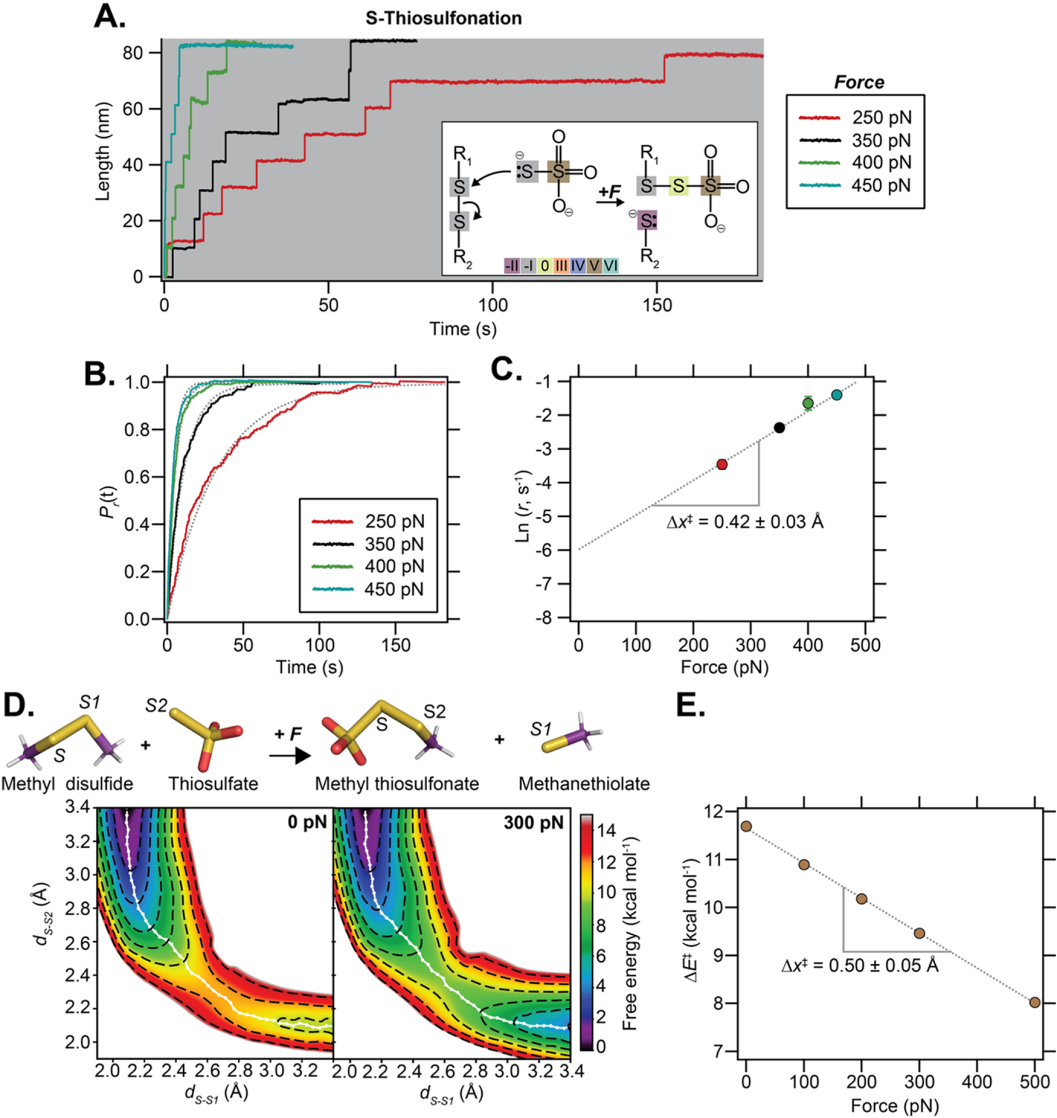
The kinetics of S-thiosulfonation under force. (A) The rate of disulfide bond reduction by thiosulfate (250 nM at pH 7.5) is accelerated by the stretching force, as observed during the second force pulse (Figure 2D) at pulling forces ranging from 250 pN to 450 pN. (B) Measurement of the rate of disulfide bond reduction by thiosulfate at four different pulling forces. At each force, we averaged and normalized 15–40 traces to obtain the probability of reduction (*P*_*r*_). The reduction rate at each force is obtained by fitting a single exponential to the average trace (dotted gray line). 250 pN *N* = 25 trajectories; 350 pN *N* = 34 trajectories; 400 pN *N* = 20 trajectories; 450 pN *N* = 16 trajectories. (C) Semilogarithmic plot of the disulfide bond reduction rate by thiosulfate as a function of the pulling force. Fits of the Arrhenius term *r*(*F*) = *r*_0_ exp­(*F*Δ*x* ^‡^/*kT*) to the experimental data give Δ*x* ^‡^ = 0.42 ± 0.03 Å (dotted gray line). (D) Energy surface for the reaction between methyldisulfide and thiosulfate obtained from quantum calculations at 0 pN (left), to which the work of force on *d*_S–S1_ was added to obtain the same surface at increasing forces, for example 300 pN (right). The white string corresponds to the average minimum energy path at 300 K. Atom labels are shown on the molecular structures shown above, which correspond to the minimized conformations in the reactant and product states, respectively. (E) The reaction energy barrier is determined at each force as the difference between the maximum value of the energy along the path and that of the first point of the path (reactant state, upper left). The force dependency allows determination of the distance to the transition state in the Bell model formalism.

To obtain an atomistic, detailed picture of how force modulates the free-energy surface of disulfide bond scission by thiosulfate, we complemented our single bond experiments with DFT calculations. These calculations enabled us to identify the height of the main energy barrier determining the reaction (Δ*E*_0_^‡^) and measure how its height is modulated by the applied force (Figure 3D). Δ*E*_0_^‡^ was determined by a systematic scan along the initial disulfide bond distance (*d*_SS_) and sulfur-nucleophile distance, which allowed us to identify the lowest energy pathway between reactants and products, and consequently the barrier (Figure S6). The energy landscape under force was then obtained by adding a work *–Fd*_SS_ contribution and then using the protocol described above to determine the force-dependent barrier Δ*E* ^‡^(*F*). Assuming a simple Bell/Arrhenius behavior, Δ*E* ^‡^(*F*) = Δ*E*_0_^‡^ – *F*Δ*x* ^‡^, these calculations resulted in a Δ*x* ^‡^ = 0.50 ± 0.05 Å (Figure 3E).

Alternatively, Δ*x* ^‡^ can be independently calculated by projecting the energy path along *d*_SS_1__ and measuring the difference in *d*_SS_1__ between reactant and product. As the top of the barrier is relatively flat, the measured Δ*x* ^‡^ slightly depends in this case on the pulling force, rapidly plateauing toward 0.51 Å (Figure S7). In both cases, the excellent agreement between the calculated and the experimental Δ*x* ^‡^ values suggest that, similar to organic thiols, the distance to the transition state of the reaction can be related to the elongation of the S–S bond at the transition state.^11^

We next focused on the reversibility of the force-induced reaction. We conjectured, using a similar interpretation to that employed to rationalize covalent S_N_2 disulfide bond rupture with organic nucleophiles,^15^ that reaction activation occurs once force lowers the energy barrier that separates products from reactants in the 1D reaction coordinate probed in the single-molecule experiments (Figure 4A). For a thermodynamically favored reaction, products have a lower free energy than reactants (Δ*G* ^0^ < 0, green region) (Figure 4A, left), and the application of force speeds up barrier crossing. However, returning products back to reactants in the absence of force is uphill, implying that the reverse reaction does not occur (red arrow). On the contrary, for a thermodynamically nonfavored reaction, the free energy of the products is higher than that of the reactants (Δ*G* ^0^ > 0) and the barrier separating both states is large (red region). In this limit case, force can lower the energy barrier by an amount such that the barrier cusp aligns with the free energy of the products (green region), in which case the reaction becomes suddenly possible (Figure 4A, right). The corollary is that, upon force withdrawal, the initially nonfavored products can suddenly transform back into reactants spontaneously (green arrow).

**Figure 4:**
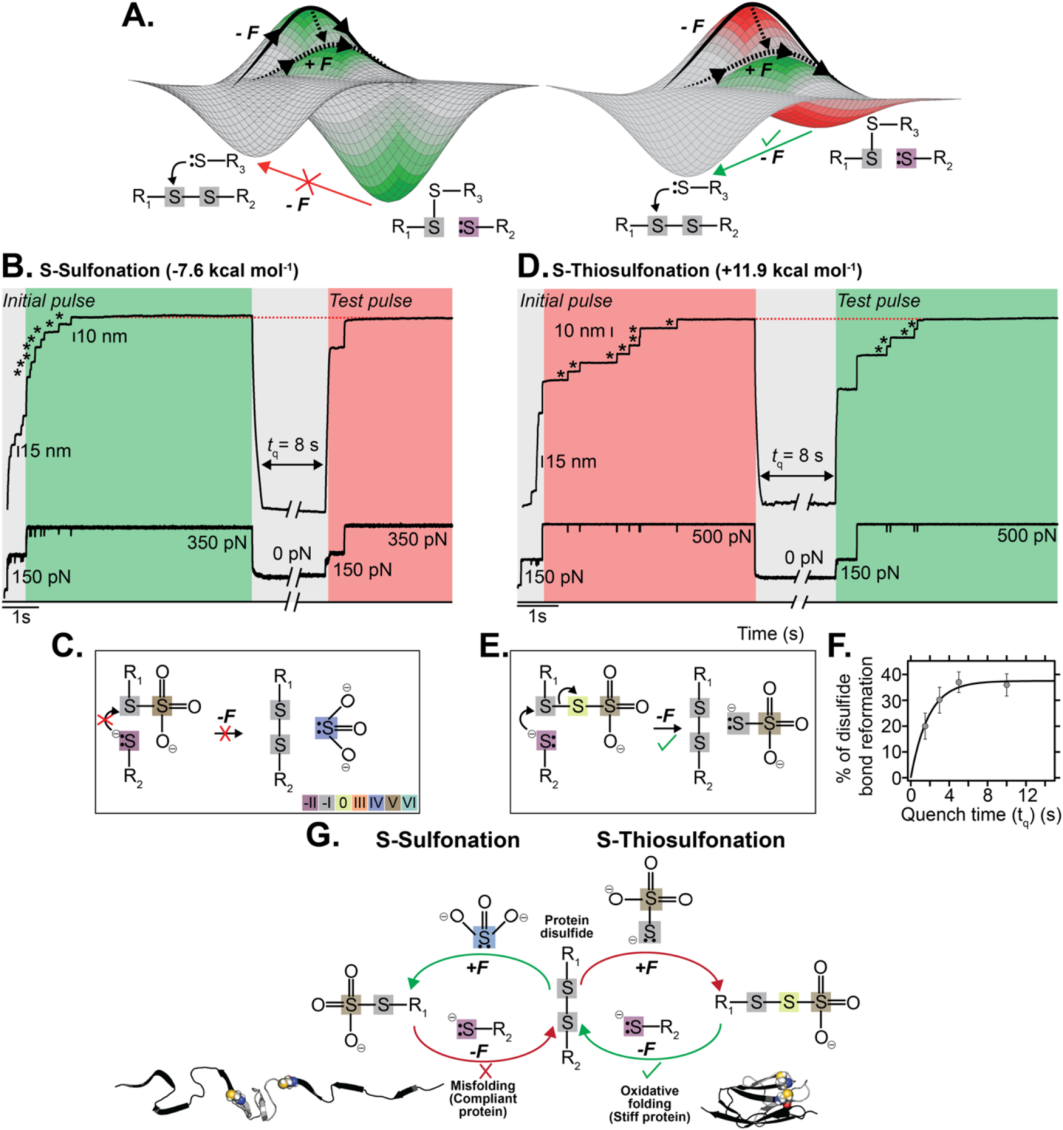
The reversibility of force-induced S-thiosulfonation. (A) Schematics of a thermodynamically favored (left) and disfavored (right) disulfide S_N_2 exchange energy surface under force. (B) Protein disulfide bond reduction by sulfite is thermodynamically favored (green region), resulting in a low-energy mixed-disulfide product that cannot reversibly reform the initial disulfide reactant in the absence of force. This is marked by the absence of ∼10 nm steps in the test pulse (red region, *N* = 18 trajectories) after a force quenching time *t*_q_ = 8 s. Dotted red line marks the full extension of the protein after the *Initial* and *Test* pulses. (C) Schematics of the reactivity defining the irreversible S-sulfonation. (D) Protein disulfide bond reduction by thiosulfate, while thermodynamically not favored (red region), is possible under force. Such a force-catalyzed reactivity renders the reaction suddenly reversible, hallmarked by the presence of ∼10 nm steps in the refolding pulse (green region) after *a* *t*_q_ = 8 s. (E) Schematics of the reversible S-thiosulfonation. (F) The percentage of protein disulfide bond reformation increases exponentially with *t*_q_, yielding a bond reformation rate *k* = 0.54 s^–1^ (*N*_1.5s_ = 10, *N*_3s_ = 23, *N*_5s_ = 28 and *N*_10s_ = 24 trajectories). (G) Summary of the implications of chemical reactivity on protein nanomechanics.

We employed our single-molecule force-quench experiments to test both potentially distinct reversibility scenarios when an individual protein disulfide bond is reduced by either the thermodynamically favored sulfite (SO_3_^2–^) or the thermodynamically impaired thiosulfate (S_2_O_3_^2–^). Using a five-force-pulse protocol (Figure 4B), we first applied an initial short pulse of 150 pN to the (Ig27_E24C–K55C_)_8_ polyprotein, resulting in the unfolding of the protein up to the disulfide bridge, which becomes solvent-exposed. A second pulse at a higher force (350 pN) triggered a ∼10 nm stepwise increase in protein length (asterisks), corresponding to the force-mediated rupture of each individual disulfide bond by the attacking sulfite. The initial two-force pulse results in a fully stretched and reduced protein containing a free cysteine thiolate and a mixed disulfide between the protein cysteine and the attacking sulfite, thus creating an S-sulfonated protein. Removal of the stretching force for *t*_q_ = 8 s triggered the collapse of the protein, potentially enabling its oxidative folding. A second test pulse, mirroring the initial pulse, probed the chemical and folding reversibility success for a given *t*_q_. The subsequent reapplication of force resulted in a protein extension devoid of any steps for any *t*_q_ (Figure 4B). The lack of ∼10 nm steps in the test pulse unambiguously demonstrated that the mixed-disulfide between sulfite and the protein cysteine is energetically very stable, thus preventing the reattack by the free protein thiols (Figure 4C), consequently inhibiting refolding. These results are in line with DFT calculations and the colorimetric Ellman’s assay; since disulfide reduction by sulfite is thermodynamically favored, the reverse reaction is necessarily impaired.

We then repeated the same refolding experiments in the presence of thiosulfate. In this case, analogous to the initial force-pulse exhibiting the unfolding and the (thermodynamically impaired) reduction of the disulfide bond under force (Figure 2D), the elongation of the protein in the test pulse followed a sequential reduction of individual disulfide bonds (∼10 nm steps) (Figure 4D). Of note, the efficiency of disulfide bond reformation depends on the time the force is quenched, *t*_q_. In this particular trajectory, the *Initial* pulse (red) exhibits 7 individual disulfide bond rupture events (marked with an asterisk), whereas the *Test* pulse (green) contains only 4 events. This implies that, for *t*_q_ = 8 s, not all disulfide bonds completely reformed, but rather 4/7 = 57%. Crucially, the presence of disulfide rupture events in the *Test* pulse fingerprints the reversibility of the S-thiosulfonation (Figure 4E). The probability of reforming the protein disulfide depends on the quenching time (*t*_q_) (Figure 4F), ultimately resulting in a successfully refolded (and reoxidized) protein. Also, in this case, the reaction reversibility can be predicted by DFT calculations. Given that disulfide reduction by thiosulfate is not thermodynamically favored (and nevertheless activated by force), the reverse reaction (i.e., the reformation of the disulfide bond from the S-thiosulfonated moiety and the neighboring thiolate) will be necessarily spontaneous, in line with our single-molecule observations.

## Discussion

We primarily used the Ig27 protein as a well-defined substrate to interrogate chemical reactivity under force. However, given the protein context where chemical reactivity takes place, our conclusions have direct implications for the relationship between (nonenzymatic) oxidative folding and protein elasticity. When post-translationally sulfonated after sulfitolysis,^23^ the mechanically stiff Ig27 titin domain turns into a compliant polypeptide devoid of mechanical stability. By contrast, upon S-thiosulfonation under force, and in the presence of a neighboring thiol in the protein structure, the mechanically stiff disulfide bonds are readily reformed, altogether rendering the Ig27 protein properly refolded and mechanically stable (Figure 4G). Consequently, disulfide cleavage with two structurally and chemically similar oxyanions gives rise to two protein forms of markedly different mechanical functions. Given the emerging role of sulfite and thiosulfate in cardiac and mitochondrial dysfunction and adverse reactions,^24^ and in aortic smooth muscle cells differentiation and stimulation of angiogenesis and vascular repair,^25^ respectively, it is tempting to hypothesize that their related post-translational modifications might be related to the gain and loss of mechanical function of the proteins at play.

## Supplementary Material



## Data Availability

All optimized structures (.xyz format) generated using both DFT functionals are available on Zenodo (10.5281/zenodo.16995322). The data that support the plots within this paper and other findings of this study are available from the corresponding author upon reasonable request.

## Notes

Experimental details of protein engineering, AFM and colorimetric experiments; DFT calculations and structures; Force-dependent energy landscapes (Figures S1–S7) (PDF)
