# Supplementary material for: Force-Triggered Thermodynamically Uphill Disulfide Reduction through Sulfur Oxidation State Control
Source: J Am Chem Soc. 2025 Oct 1;147(41):37701–7. doi: 10.1021/jacs.5c13084 (PMC12532186; doi:10.1021/jacs.5c13084)
Supplement: Supplementary file 1 [file ja5c13084_si_001.pdf]

Supplementary Online Information for:

Force-triggered thermodynamically-uphill disulfide reduction through sulfur oxidation state control

Marc Mora[1,2], Georgia Cohen[1,2], William Cranton[1,2], Olaia Anton[4], Amy E. M. Beedle[3], Guillaume Stirnemann[4] and Sergi Garcia-Manyes[1,2 ¶]

[1]Department of Physics, Randall Centre for Cell and Molecular Biophysics, Centre for the Physical Science of Life and London Centre for Nanotechnology, King's College London, Strand, WC2R 2LS, London, UK

[2]Single Molecule Mechanobiology Laboratory, The Francis Crick Institute, 1 Midland Road, London, NW1 1AT, UK

[3]Department of Physics and Centre for the Physical Science of Life, King's College London, Strand, WC2R 2LS, London, UK

[4]CPCV, Département de Chimie, École Normale Supérieure, PSL University, Sorbonne University, CNRS, 75005 Paris, France

[¶]Corresponding author: sergi.garcia-manyes@kcl.ac.uk

Materials and methods

Protein engineering

The Ig27($_{E24C-K55C}$)$_8$ polyprotein was subcloned using the BamHI, BglII and kpn1 restriction sites. The polyprotein construct was then cloned into the pQE80L (Qiagen) expression vector, and transformed into the BLR(DE3) *Escherichia Coli* expression strain. Cells were grown at 37 ºC in Luria-Bertani (LB) broth supplemented with 100 µg L^{-1} of ampicillin. Upon an OD_{600} of 0.6, 1 mM of IPTG was used to induce the cultures, which were then incubated overnight at 20 ºC. The cells were then harvested and disrupted using a French Press. The proteins from the lysate were purified twice, first by metal affinity chromatography on Talon resin (Clontech) and then by gel-filtration using a Superdex 200 10/300 GL column (GE Biosciences).

Colorimetric assay

Colorimetric Ellman's assays were conducted by incubating freshly prepared solutions of sodium sulfite and sodium thiosulfate with 5,5'-dithio-bis-(2-nitrobenzoic acid), DTNB. DTNB is an organic compound that contains a symmetric disulfide bond that can be readily reduced by a nucleophile, yielding two different molecules, a mixed disulfide and a 2-nitro-5-thiobenzoic acids (TNB). TNB is responsible for the characteristic yellow colour of the Ellman's solution, serving as a direct reporter of disulfide bond reduction. DTNB was prepared at a final concentration of 1 mM with 3 mL of ethanol at pH 7.5 in PBS buffer. Solutions of the two oxyanions were prepared at a final concentration of 20 mM. The blank solution was prepared by mixing 1:1 DTNB solution with PBS buffer. Final solutions of inorganic oxyanions with DTNB were prepared by mixing them in vials at 1:1 ratio (v/v). All reactions were performed at room temperature. Absorbance measurements were conducted using a JENWAY 6305 spectrophotometer at 412 nm on the same solutions contained in the aforementioned vials.

Single-molecule force-clamp spectroscopy experiments

Single-molecule force-clamp using the Atomic Force Microscopy (AFM) were conducted at room temperature using both a home-made set-up and a commercial Luigs and Neumman force spectrometer as described previously(*1*). In a nutshell, the measuring sample was prepared by depositing 0.5-1.5 µL of fresh protein (at a concentration of 0.5-5 mg mL^{-1}) in PBS-based buffer solution containing the desired concentration of oxyanion onto freshly evaporated gold cover slides. Gold cover slides were plasma-cleaned before each experiment for 10 min at the highest intensity. Prior to each experiment, the AFM cantilever (Si_3N_4 Bruker MLCT-AUHW) was individually calibrated using the equipartition theorem, giving rise to a typical spring constant between 12 to 20 pN nm^{-1}. Each individual polyprotein was stretched by initially pushing the cantilever onto the surface (500-2200 pN for 0.1 to 2 s) to promote adhesion between the protein and the cantilever. The piezoelectric actuator was then retracted to produce a set deflection (*i.e.*, the applied force), which was held constant throughout the entirety of the pulling trajectory. An external, active electronic feedback mechanism maintained a constant force while the protein extension was monitored. The force feedback is based on a Proportional, Integral and Differential amplifier (PID) whose output was fed to the piezoelectric positioner. In every experiment, the feedback response was limited to 1-3 ms. All force-clamp traces were filtered using a Pole Bessel filter at 1 kHz.

For protein disulfide bond reduction kinetic experiments, the Ig27($_{E24C-K55C}$)$_8$ polyprotein was first unfolded at 150 pN for 0.5 s. The force was subsequently raised up to 350 pN (for sulfite) and to a force value spanning 250- 450 pN (thiosulfate) and left constant for a long,

variable period of time to capture the full kinetics of disulfide reduction for each oxyanion. For sulfite-based disulfide bond reduction, different concentrations of sulfite were used at each pH: pH=6.5 350 mM; pH=7.5 250 mM and pH=8.5 100 mM. For S-thiosulfonation-based reductions measurements, experiments were performed at 250 mM concentration of thiosulfate.

For disulfide bond reformation experiments, the polyprotein was first unfolded at 150 pN for 0.5 s, followed by a high force pulse (varying between 300 to 500 pN depending on the oxyanion) for ~6 s. After a quench in force, a mirroring first two-force pulse was applied. Both oxyanions (Sodium Sulfite, >98 %, Sigma Aldrich; Sodium thiosulfate>98 %, Sigma Aldrich) were prepared in a sodium phosphate buffer (50 mM sodium phosphate (Na_2HPO_4 and NaH_2PO_4), 150 mM NaCl), and the pH was accordingly adjusted. Each solution was filtered through a 0.2 um membrane before each experiment. Solutions were prepared fresh each experiment day.

Data analysis

All data was recorded and analysed using custom software written in Igor Pro 8.0 (Wavemetrics). For the disulfide kinetics studies, only recordings showing the signature of at least 5 unfolding events (~15 nm steps) followed by 5 reduction events (~10 nm steps) and a maximum of 8 unfolding events and followed by 8 reduction events were considered for analysis. At each particular force, proteins showing disulfide bond reduction events had to remain attached for a long time (compared to their reduction rate) before detaching from either the cantilever tip or the gold surface, to avoid skewing the disulfide bond rupture kinetics (*2*,*3*). Specifically, the minimum cut-off detachment time for thiosulfate reduction was set at 100 s (250 pN), 50 s (350 pN), 20 s (400 pN) and 14 s (450 pN). To obtain the reduction rate in each case, we summed and normalized the reduction trajectories at each particular force. We then fitted the resulting summed trace with a single exponential to obtain the reduction rate and the standard error by bootstrapping a set of randomly selected traces. The reduction rates were then corrected by the concentration used in each condition. For protein disulfide bond reformation experiments, only trajectories with a minimum of 5 and a maximum of 8 rupture events in the first high-force pulse were considered. Moreover, trajectories were only analysed if the polyprotein was extended to the same total length in the initial and refolding pulses. Standard deviation for each refolding fraction was estimated through the bootstrap method.

Density Functional Theory (DFT) calculations

Electronic structure calculations using the density functional theory (DFT) were performed with the Orca software (*4*), using the ‘direct method’ instead of a thermodynamic cycle, which was shown to lead to successful results for a series of thiol compounds (*5*) and in our previous study (*6*). In such a strategy, all structures were minimised in implicit water directly. Frequency calculations were then performed, and the reported standard free energies contain contributions from the 0 K energy, the zero-point energy, thermal corrections at 298 K, and the solvation-free energy (from a conductor-like polarizable continuum model, CPCM model) at 298 K. We determined atomic charges using the Hirshfeld charge analysis. For each oxyanion, we estimated the energies of two different species, the oxyanion and the heterodimer containing a mixed disulfide with methylthiol.

We optimized initial geometries for reactants and products using autode (*7*) using the PBE0 functionnal (*8*) with D3 dispersion corrections with Becke-Johnson damping (*9*), the def2-SVP basis set (*10*) and implicit solvation in water using the CPCM model.

We then evaluated two different combinations of DFT functionals and basis sets for refinements of geometries and frequency calculations. We performed calculations using the B3LYP functional (*11*,*12*), the def2-TZVP basis set, still in CPCM water. We repeated the calculations with the M06-2X functional (*13*), a hybrid meta-GGA functional known for its reliable performance, along with diffuse ma-def2-TZVP basis set (*14*) in CPCM water. All optimized structures (.xyz format) are available on Zenodo (10.5281/zenodo.16995322).

To obtain the force-dependent barrier $\Delta E^{\ddagger}(F)$, we proceeded as follows. We first performed a 2D scan along the initial disulfide bond distance (d_{SS}) and sulfur-nucleophile distance both between 1.9 Å and 3.4 Å by increments of 0.15 Å, resulting in 100 calculations, using the *scan* feature of Orca: each configuration was optimized and single point calculations performed using B3LYP-D3 and the def2-SVP basis set. Each optimized structure was then optimized again at the M06-2X-D3 level, using the same basis set. The energy landscape at zero force was then obtained by interpolating the obtained energies. The minimum energy pathway connecting reactants and products was determined by relaxing a dynamic string at 300 K (*15*) on the surface. The effect of force was obtained by subtracting $-Fd_{SS}$ to each point on the surface, and then recalculating the barrier with a new string.

A.

S-Sulfonation (SO_3^{2-})

**Functional**	**Δ*G*° rupture (kcal mol^{-1})**	**Sulfur charge**
B3YLP-D3/ def2-TZVP/ CPCM water	**-14.4**	**-0.055**
M062X-D3/ ma-def2-TZVP/ CPCM water	**-7.6**	**-0.068**

B.

B3YLP-D3/ def2-TZVP/ CPCM water

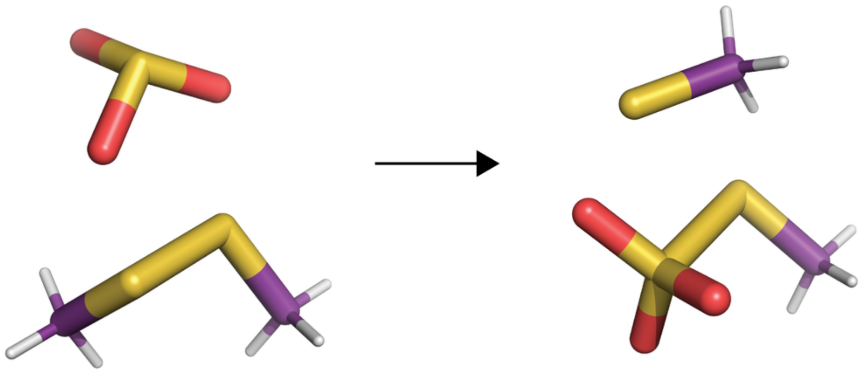

M062X-D3/ ma-def2-TZVP/ CPCM water

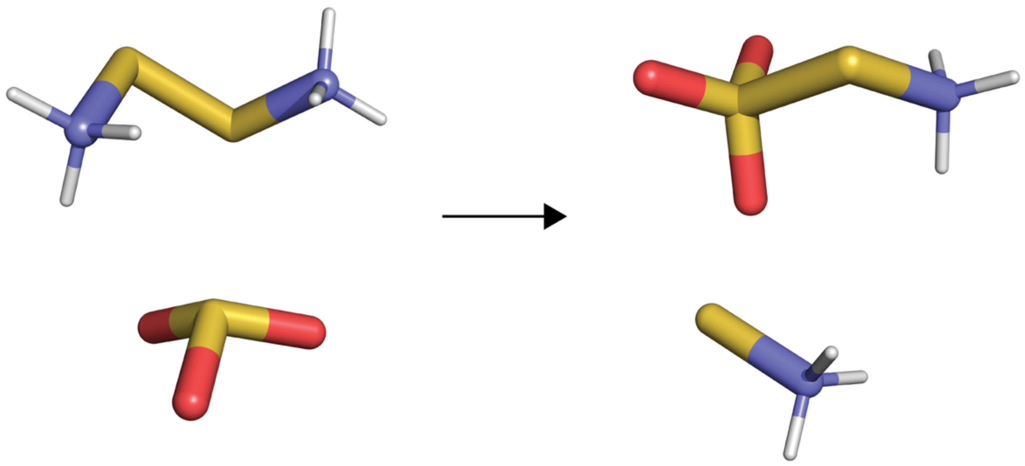

Supplementary Figure S1. Comparison of S-Sulfonation reactivity calculated using two different combinations of DFT functionals and basis sets. A. Comparison of the DFT calculations of the standard free-energy and sulfite sulfur's charge associated with disulfide rupture of two methyl thiols by sulfite using two different functionals. **B.** Stick representation of the structures used in the DFT calculations using both functionals.

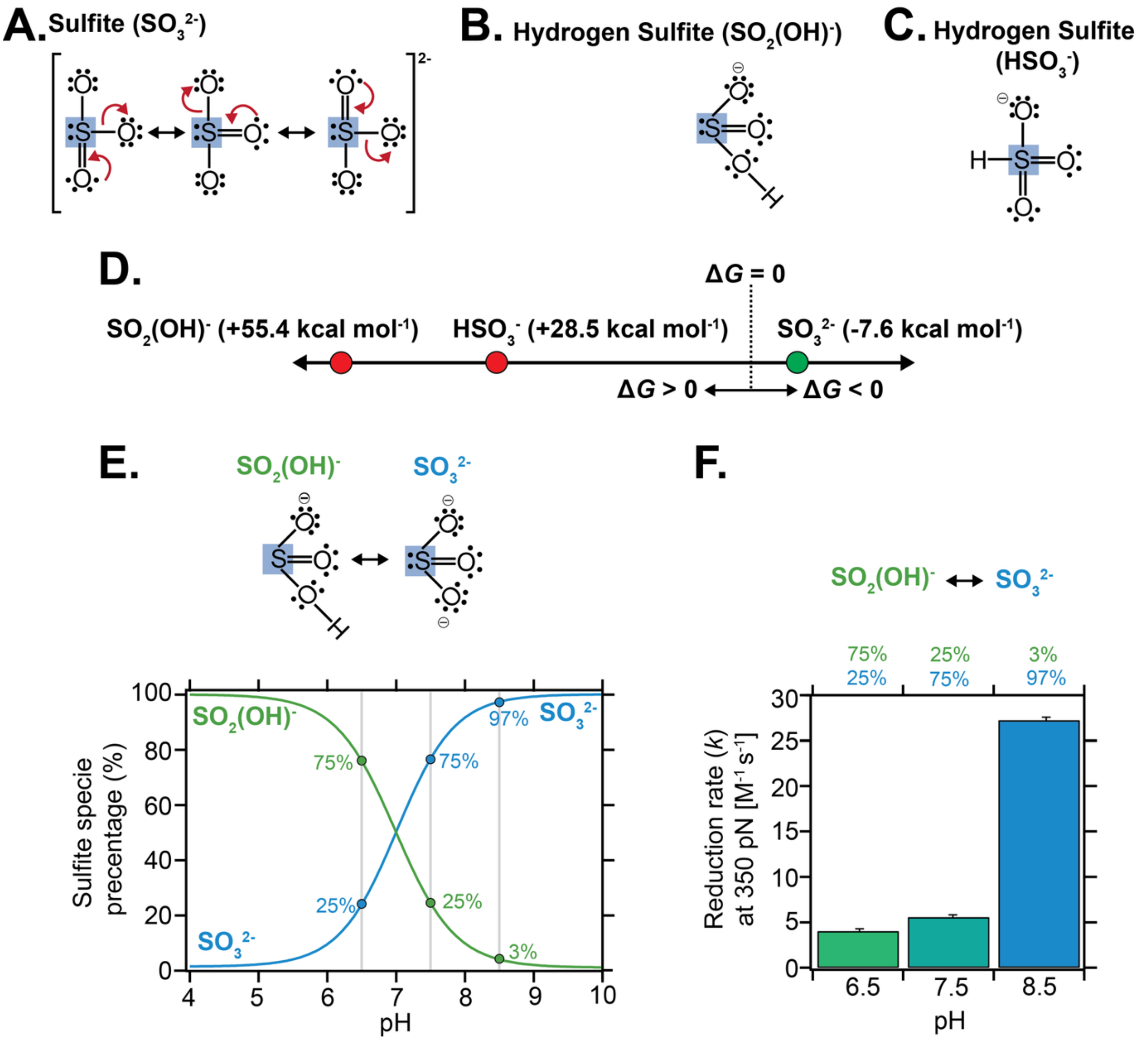

Supplementary Figure S2. Impact of the sulfite's chemistry on the reduction rate of a protein disulfide bridge. A. Sulfite anion and its three different resonant structures, which enhance sulfur's overall nucleophilicity. **B-C.** The two different possible structures of hydrogen sulfite. **D.** DFT calculations of the standard free energy (M06-2X functional with the ma-def2-TZVP basis set in CPCM implicit solvent) associated with the cystine disulfide rupture by the three possible sulfite forms depicted in A-C. **E.** pH dependency of sulfite and the different proportion of species present at a given pH. **F.** Normalized reduction rates of the protein's disulfide by sulfite under different pH conditions (pH 6.5 *N*=8, pH 7.5 *N*=15, pH 8.5 *N*=21 reduction traces).

A. **O-Hydrogensulfonation (HSO_3^-)**

Functional	ΔG^o rupture (kcal mol^{-1})	Sulfur charge
B3YLP-D3/ def2-TZVP/ CPCM water	**+28.8**	**-0.458**
M062X-D3/ ma-def2-TZVP/ CPCM water	**+28.5**	**-0.464**

B.

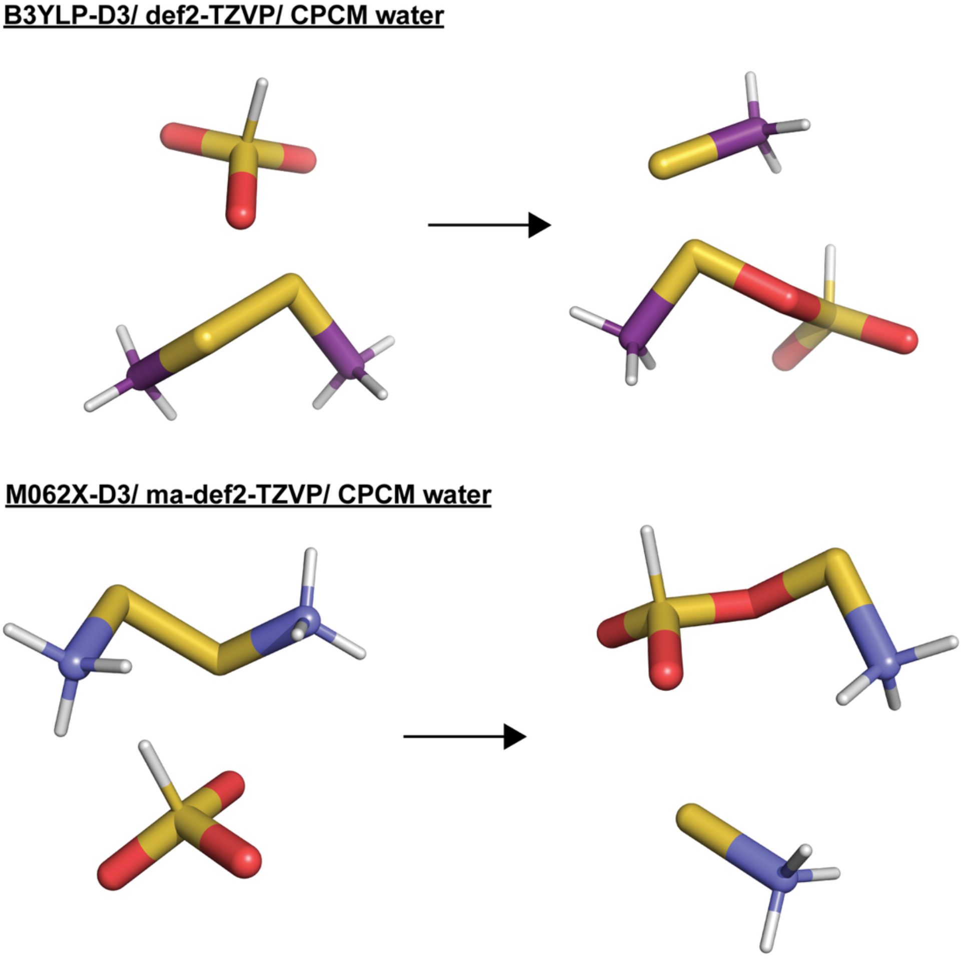

Supplementary Figure S3. Comparison of O-Hydrogensulfonation reactivity calculated using two different combinations of DFT functionals and basis sets. A. Comparison of the DFT calculations of the standard free-energy and hydrogensulfite sulfur's charge associated with disulfide rupture of two methyl thiols by hydrogensulfite using two different functionals. **B.** Stick representation of the structures used in the DFT calculations using both functionals.

A. **S-Hydrogensulfonation ($SO2(OH)^-$)**

Functional	ΔG^o rupture (kcal mol^{-1})	Sulfur charge
B3YLP-D3/ def2-TZVP/ CPCM water	**+56.7**	**0.335**
M062X-D3/ ma-def2-TZVP/ CPCM water	**+55.4**	**0.336**

B.

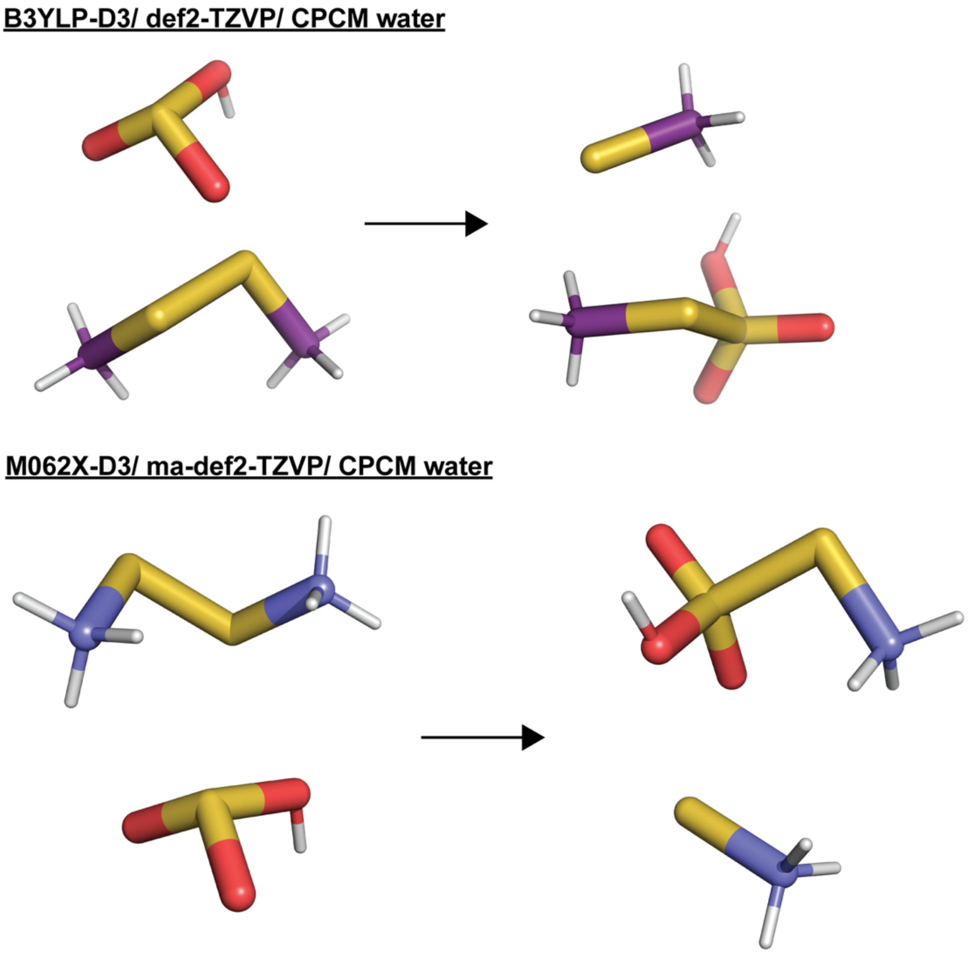

Supplementary Figure S4. Comparison of S-Hydrogensulfonation reactivity calculated using two different combinations of DFT functionals and basis sets. A. Comparison of the DFT calculations of the standard free-energy and hydrogensulfite sulfur's charge associated with disulfide rupture of two methyl thiols by hydrogensulfite using two different functionals. **B.** Stick representation of the structures used in the DFT calculations using both functionals.

A.

S-Thiosulfonation ($S_2O_3^{2-}$)

**Functional**	**ΔG^{o} rupture (kcal mol^{-1})**	**Sulfur charge**
B3YLP-D3/ def2-TZVP/ CPCM water	**+9.4**	**-0.728**
M062X-D3/ ma-def2-TZVP/ CPCM water	**+11.9**	**-0.713**

B.

<u>B3YLP-D3/ def2-TZVP/ CPCM water</u>

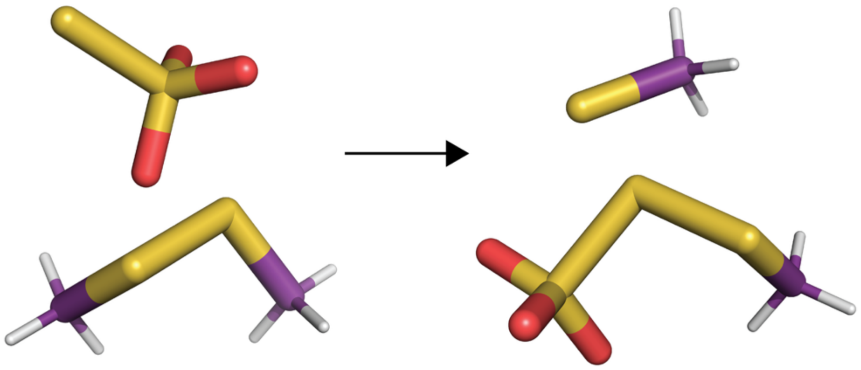

<u>M062X-D3/ ma-def2-TZVP/ CPCM water</u>

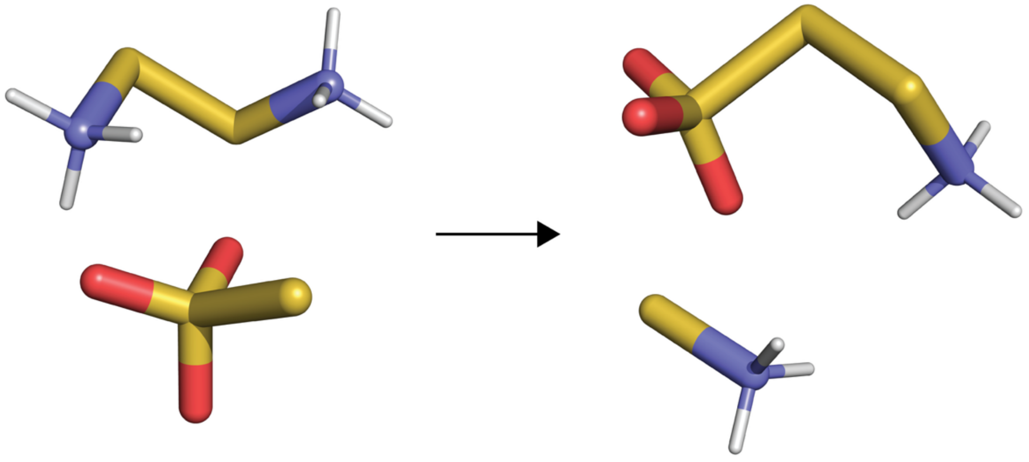

Supplementary Figure S5. Comparison of S-Thiosulfonation reactivity calculated using two different combinations of DFT functionals and basis sets. A. Comparison of the DFT calculations of the standard free-energy and thiosulfate sulfur's charge associated with disulfide rupture of two methyl thiols by thiosulfate using two different functionals. **B.** Stick representation of the structures used in the DFT calculations using both functionals.

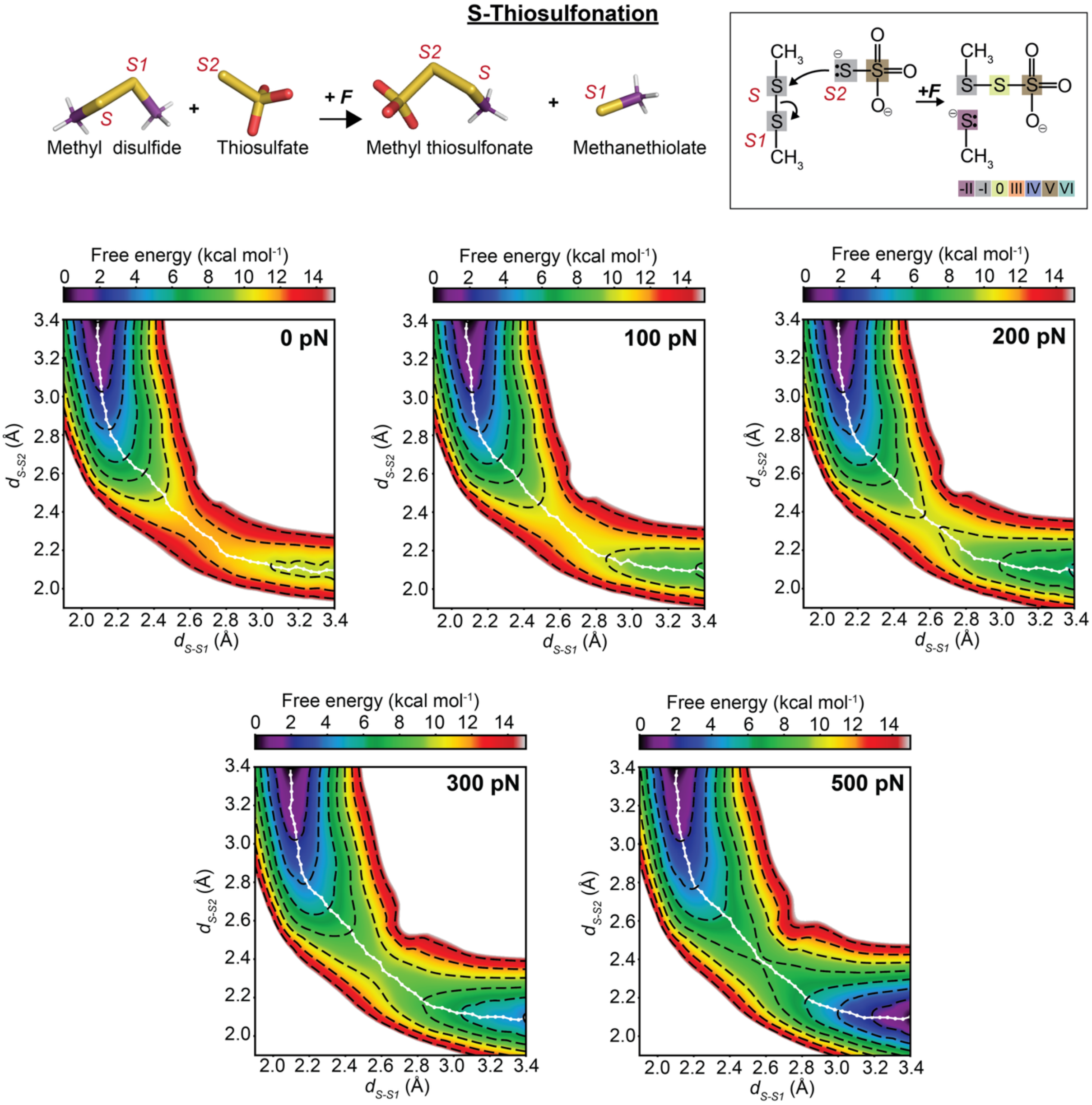

Supplementary Figure S6. Force dependent energy landscapes for the reduction of methyldisulfide by thiosulfate. Energy surface for the reaction between methyldisulfide and thiosulfate obtained from quantum calculations at 0 pN (upper left), to which the work of force on $d_{S\text{-}S1}$ was added to obtain the same surface at increasing forces (other panels). The white string corresponds to the average minimum energy path at 300 K. Atom labels are shown on the molecular structures.

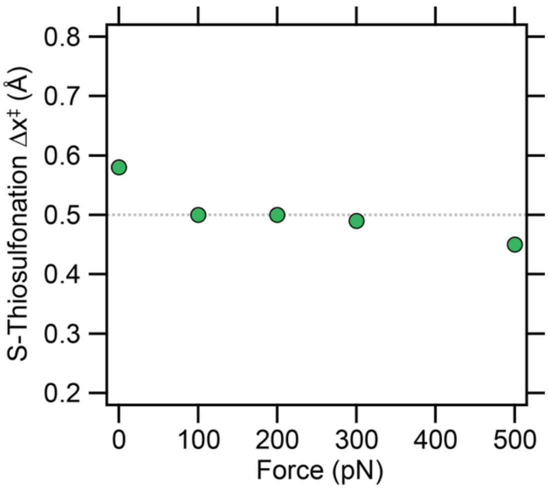

Supplementary Figure S7. Estimating S-thiosulfonation $\Delta x^{\ddagger}$ from energy proflies under force. While $\Delta x^{\ddagger}$ can be calculated from the force-dependence of the energy barrier using the Bell model, $\Delta x^{\ddagger}$ can be independently calculated by projecting the energy path along d_{SS} and measuring the difference in d_{SS} between reactant and product. As the top of the barrier is relatively flat, the measured $\Delta x^{\ddagger}$ slightly depends in this case on the pulling force, rapidly plateauing towards ~0.5 Å**.**

References

1. Popa I, Kosuri P, Alegre-Cebollada J, Garcia-Manyes S, Fernandez JM. Force dependency of biochemical reactions measured by single-molecule force-clamp spectroscopy. *Nature Protocols*. **2013**;8:1261–1276. https://doi.org/10.1038/nprot.2013.056
2. Garcia-Manyes S, Bruji&Cacute J, Badilla CL, Fernández JM. Force-Clamp spectroscopy of Single-Protein monomers reveals the individual unfolding and folding pathways of i27 and ubiquitin. *Biophysical Journal*. **2007**;93:2436–2446. https://doi.org/10.1529/biophysj.107.104422
3. Hermans RI. Probability of Observing a Number of Unfolding Events while Stretching Polyproteins. *Langmuir*. 2014;30(29):8650–8655. https://doi.org/10.1021/la501161p
4. Neese F. Software update: The ORCA program system—Version 5.0. *Wiley Interdisciplinary Reviews Computational Molecular Science*. **2022**;12. https://doi.org/10.1002/wcms.1606
5. Thapa B, Schlegel HB. Density Functional theory calculation of PKA's of thiols in aqueous solution using explicit water molecules and the polarizable continuum model. *The Journal of Physical Chemistry A*. **2016**;120:5726–5735. https://doi.org/10.1021/acs.jpca.6b05040
6. Beedle AEM, Mora M, Davis CT, Snijders AP, Stirnemann G, Garcia-Manyes S. Forcing the reversibility of a mechanochemical reaction. *Nature Communications*. **2018**;9. https://doi.org/10.1038/s41467-018-05115-6
7. Young TA, Silcock JJ, Sterling AJ, Duarte F. AUTODE: Automated Calculation of Reaction Energy Profiles— application to organic and organometallic reactions. *Angewandte Chemie International Edition*. **2020**;60:4266–4274. https://doi.org/10.1002/anie.202011941
8. Adamo C, Barone V. Toward reliable density functional methods without adjustable parameters: The PBE0 model. *The Journal of Chemical Physics*. **1999**;110:6158–6170. https://doi.org/10.1063/1.478522
9. Grimme S, Ehrlich S, Goerigk L. Effect of the damping function in dispersion corrected density functional theory. *Journal of Computational Chemistry*. **2011**;32:1456–1465. https://doi.org/10.1002/jcc.21759
10. Hellweg A, Rappoport D. Development of new auxiliary basis functions of the Karlsruhe segmented contracted basis sets including diffuse basis functions (def2-SVPD, def2-TZVPPD, and def2-QVPPD) for RI-MP2 and RI-CC calculations. *Physical Chemistry Chemical Physics*. **2014**;17:1010–1017. https://doi.org/10.1039/c4cp04286g
11. Becke AD. Density-functional exchange-energy approximation with correct asymptotic behavior. *Physical Review A, General Physics*. 1988;38(6):3098–3100. https://doi.org/10.1103/physreva.38.3098
12. Lee C, Yang W, Parr RG. Development of the Colle-Salvetti correlation-energy formula into a functional of the electron density. *Physical Review B, Condensed Matter*. **1988**;37:785–789. https://doi.org/10.1103/physrevb.37.785
13. Zhao Y, Truhlar DG. Exploring the limit of accuracy of the global hybrid meta density functional for Main-Group thermochemistry, kinetics, and noncovalent interactions. *Journal of Chemical Theory and Computation*. **2008**;4:1849–1868. https://doi.org/10.1021/ct800246v
14. Zheng J, Xu X, Truhlar DG. Minimally augmented Karlsruhe basis sets. *Theoretical Chemistry Accounts*. **2010**;128:295–305. https://doi.org/10.1007/s00214-010-0846-z
15. E W, Ren W, Vanden-Eijnden E. String method for the study of rare events. *Physical Review B, Condensed Matter*. **2002**;66. https://doi.org/10.1103/physrevb.66.052301
